# High-dose naloxone formulations are not as essential as we thought

**DOI:** 10.1186/s12954-024-00994-z

**Published:** 2024-05-13

**Authors:** Paige M. Lemen, Daniel P. Garrett, Erin Thompson, Megan Aho, Christina Vasquez, Ju Nyeong Park

**Affiliations:** 1 Tennessee Harm Reduction, 1989 Madison Avenue, 7, Memphis, TN 38104 USA; 2 University of Tennessee Health Science Center, Memphis, TN USA; 3 Harm Reduction Innovation Lab, Rhode Island Hospital, Providence, RI USA; 4 The Warren Alpert Medical School, Brown University, Providence, RI USA

**Keywords:** Naloxone, Opioid overdose, Literature review

## Abstract

Naloxone is an effective FDA-approved opioid antagonist for reversing opioid overdoses. Naloxone is available to the public and can be administered through intramuscular (IM), intravenous (IV), and intranasal spray (IN) routes. Our literature review investigates the adequacy of two doses of standard IM or IN naloxone in reversing fentanyl overdoses compared to newer high-dose naloxone formulations. Moreover, our initiative incorporates the experiences of people who use drugs, enabling a more practical and contextually-grounded analysis. The evidence indicates that the vast majority of fentanyl overdoses can be successfully reversed using two standard IM or IN dosages. Exceptions include cases of carfentanil overdose, which necessitates ≥ 3 doses for reversal. Multiple studies documented the risk of precipitated withdrawal using ≥ 2 doses of naloxone, notably including the possibility of recurring overdose symptoms after resuscitation, contingent upon the half-life of the specific opioid involved. We recommend distributing multiple doses of standard IM or IN naloxone to bystanders and educating individuals on the adequacy of two doses in reversing fentanyl overdoses. Individuals should continue administration until the recipient is revived, ensuring appropriate intervals between each dose along with rescue breaths, and calling emergency medical services if the individual is unresponsive after two doses. We do not recommend high-dose naloxone formulations as a substitute for four doses of IM or IN naloxone due to the higher cost, risk of precipitated withdrawal, and limited evidence compared to standard doses. Future research must take into consideration lived and living experience, scientific evidence, conflicts of interest, and the bodily autonomy of people who use drugs.

## Background

Opioid use disorder affects more than 2.1 million individuals in the United States [1]. Persistent opioid use can leave individuals with opioid dependency resulting in daily opioid use, despite potential medical and social consequences, the most significant of which is overdose [2, 3]. Opioid overdose occurs when opioids bind to and activate opioid receptors and suppress breathing rate below that which is required to maintain consciousness [4]. If suppression is continued for an extended time, health complications including death can occur.

Opioid overdose, however, is reversible if a bystander identifies an overdose in progress and administers naloxone hydrochloride (hereafter, naloxone) quickly [1, 2]. Naloxone is an opioid antagonist medication with a stronger binding affinity for opioid receptors than heroin or fentanyl. When administered, it “knocks opioids off” the opioid receptors in the central or peripheral nervous system and binds without activation, thereby reversing both intentional (i.e. analgesia, euphoria) and unintentional (i.e. respiratory depression, coma) effects of the used opioid [3, 4]. Naloxone administered by community members (bystanders, friends, family, etc.) has proven successful in reversing opioid overdoses in 75–100% of cases [5]. It is considered safe at the recommended doses to opioid-naive persons as well [5].

Naloxone is primarily available to the public through free community-based programs, though it is also available through provider prescriptions and as an over-the-counter medication at pharmacies. Community health workers, pharmacists, and medical professionals distributing naloxone often also provide training on overdose recognition and response, however, some organizations also incorporate a train-the-trainer model to enable community members to share these skills in their networks.

Community based overdose response can occur anywhere individuals use substances including private homes and public spaces (restaurants, shopping centers, laundromat, etc.) [6]. The currently recommended response protocol is a five step process: (1) checking for signs of opioid overdose, such as unconsciousness, slow or absent breathing, pale and clammy skin, and slow or no heartbeat, (2) calls emergency medical services (EMS) to ensure timely medical attention, (3) administer naloxone, (4) clear the airways to perform rescue breathing to help provide oxygen to the body, (5) administer additional naloxone if the individual does not regain consciousness and respiration [7]. An additional strategy to support someone experiencing overdose is to administer oxygen, however oxygen should not be used as the sole treatment method especially if breathing has ceased [8–10]. Many factors determine the amount of naloxone needed including type, amount, half-life and method of opioid use, tolerance levels, health status, and naloxone administration route [11–13].

While overdose detection and response is straightforward, the general population has an ingrained fear of and stigma towards PWUD. Many social and environmental factors contribute to the fear including lack of understanding, political beliefs, personal experiences, dissemination of misinformation, criminalization, and the ‘war on drugs’ mentality [14–16]. Oftentimes, individuals who use recreationally for personal, constructive purposes, and in a manner characterized by safety and responsibility, remain largely invisible within media representation and public forums [17]. Instead, the focus tends to prioritize individuals with SUD, those who overdose, or those solely in abstinence-based recovery. This biased depiction results in an altered portrayal of the effects and risks associated with recreational drug use [18] and provides opportunities for reinforcing racial, gender, and class stereotypes pertaining to drug users [18, 19]. Misunderstandings and socially reinforced biases result in misunderstandings of and opposition towards harm reduction strategies [20, 21]. In the context of naloxone, the misunderstandings can be seen in the altered portrayal of the effects and risks associated with naloxone use for overdose reversal [21–23]. It also results in sidelining of people with lived and living experience when exploring new developments regarding appropriate naloxone dose and administration.

Opioid use is a complex issue that is influenced by a variety of factors, such as social, economic, environmental, and other determinants of health. People who use drugs (PWUD) should not be delineated solely by their drug consumption, but rather recognized as multifaceted individuals with distinct requirements and aspirations. Therefore, adopting an impartial and non-judgmental approach is imperative when addressing the subject of drug use and, consequently, the application of naloxone.

Another consequence of the criminalization of non-prescribed opioid use is that it forces individuals who are dependent on opioids to use the unregulated illicit drug supply. Lack of regulatory standards results in a market fraught with impurities including both filler and undesired illicit substances. Notably, fentanyl has overtaken the heroin supply as the predominantly available opioid and has been found in non-opioid substance samples [24–26]. Fentanyl and its analogs are short acting opioids with exceptionally high potency when compared to other opioids like heroin and morphine [24]. Consequently, individuals who are exposed to fentanyl unintentionally or intentionally or at a higher purity level than anticipated are particularly vulnerable to overdose [27].

According to provisional data from the Centers for Disease Control and Prevention (CDC), there were more than 100,000 drug overdose deaths in the United States in 2021[28]. This concern for high overdose rates and drug poisoning severity provides theoretical justification for the proposal of higher doses of naloxone formulations. Nevertheless, this theoretical foundation lacks input from the ground level experts: PWUD, harm reduction workers, and other relevant groups possessing firsthand experience and direct involvement with drug users. These groups possess unique experiences and knowledge that most researchers and medical providers do not regularly have access to, including in the development of high-dose naloxone formulations.

Two previous literature reviews have been conducted on this topic: Moe and colleagues conducted a systematic review of overdoses (n = 26,660) in North America and Europe through 2018. They found that although higher initial and cumulative naloxone doses were being used by lay and healthcare responders for overdoses presumed to be fentanyl or another synthetic opioid, a cumulative total dose of 4 mg of naloxone (e.g., two doses of standard IN) was sufficient in 97% of presumed fentanyl/potent opioid cases [29]. Abdelal et al. [30] recently examined the use of two or more naloxone doses, however the authors received consultancy fees or stock options from Hika Pharmaceuticals, which manufactures the high-dose naloxone formulations product Kloxxado. The implementation of naloxone programs remains an imprecise science despite the reliability of naloxone in reversing opioid overdose. The emergence of newer formulations necessitates close examination of scientific research. Accordingly, this literature review aims to improve our understanding of how often more than two doses of IM and IN naloxone are needed to reverse a fentanyl overdose and whether promoting high-dose naloxone formulations is an optimal and necessary solution for community-led overdose response.

### Naloxone options in the U.S.

There are currently three U.S. Food and Drug Administration (FDA)-approved administration routes of naloxone that are available: injectable intramuscular (IM), intravenous (IV), and intranasal spray (IN). Only IM and IN formulations are currently used in lay person response. Subcutaneous auto-injectors were used previously but have been discontinued [31, 32]. IM naloxone solution has traditionally been provided in kits of two 1-mL vials containing a 0.4 mg/mL solution [33]. 10-mL vials containing 0.4 mg/mL are also available but are not readily accessible in IM naloxone kits provided to PWUD [34, 35]. IN naloxone is also provided in two-unit kits with a 4 mg/0.1 ml naloxone solution pre-loaded into an atomizer that is ready to use intranasally. Regardless of the exact route of administration, naloxone can be easily administered by a lay bystander as an intervention for overdose [16].

The FDA has continued to support high-dose naloxone formulations (high-dose naloxone formulations) such as Kloxxado (double the dosage of standard IN) and Zimhi (25 times higher dose than generic IM) [17], despite the voiced concerns of harm reduction workers and others with lived experience of using naloxone to reverse an overdose [36, 37]. This is in part due to concerns that the high potency of fentanyl could require higher doses of naloxone to be effective and the assumption that a single high dose formulation is preferred over multiple doses of the typical formulation. Having input on drug policy and research from people with lived and living experiences of drug use is invaluable because it provides a more comprehensive understanding of the complexities and realities of drug use. Inclusion results in policies and research that are informed by the perspectives and needs of those directly affected, leading to more effective and equitable outcomes [38]. However, the historical exclusion of these individuals can be attributed, in part, to the pervasive stigma surrounding drugs and PWUD [39].

The U.S. currently has seven overdose reversal products that contain naloxone described both below and in Table 1.

**Table 1:** Naloxone products available in the United States

Image	Original Approval Date	Brand or manufacturer name	Route of Administration	Dosage	Relative dosage compared to standard dose	Cost (as of July 2023)	Over the counter
	Oct 1985	*Hospira (generic)*	IM/IV	0.4 mg/1.0 ml	Reference (IM)	$15—40/ 2 units	No
	Nov 2015	Narcan	IN	4 mg/0.1 ml	Reference (IN)	$130—145/ 2 units	Yes
	Apr 2019	Teva generic nasal spray	IN	4 mg/ 0.1 ml	1x	$20—92/ 2 units	No
	Oct 2021	Zimhi	IM	5 mg/ 0.5 ml	25x	$131- 145/ 2 units	No
	Apr 2021	Kloxxado	IN	8 mg/ 0.1 ml	2x	$131—145/ 2 units	No
	Jun 2022	*Perrigo (Generic)*	IN	4 mg/0.1 ml	1X	$20—92/ 2 units	No
	Mar 2023	Amphastar	IN	4 mg/0.1 ml	1x	$30–60/ 2 units	No

All prices were retrieved from https://www.goodrx.com/ in July 2023

Generic injectable naloxone is one of the most popular formulations supplied to PWUD [40, 41]. It comes in 1 mL vials of 0.4 mg/mL concentration [33]. It can be utilized in any method of administration, including intramuscular (IM), and intravenous (IV) routes, as well as intranasally (IN) via an atomizer. NARCAN® Is the most well-known brand name for naloxone nasal spray. It comes with a 0.1 mL pre-packaged solution that contains 4 mg/0.1 mL of naloxone. This specific brand’s pre-packaged administration tool only allows for naloxone to be administered intranasally. The generic counterparts to NARCAN® are the Teva and Perrigo generic nasal sprays which have chemically identical active ingredients and concentrations. Kloxxado® is a newer naloxone nasal spray that also comes with double the dose of NARCAN. It comes with a 0.1 mL pre-packaged solution that contains 8 mg/0.1 mL of naloxone. Identical to NARCAN, this specific brand’s pre-packaged administration tool also only allows for naloxone to be administered intranasally. Zimhi is a brand name autoinjector that comes pre-loaded with 0.5 mL of 5 mg/0.5 mL naloxone. This syringe can only be administered intramuscularly (IM). Lastly, Amphastar® Prefilled Naloxone Syringes come in 2 mL, with 1 mg/mL naloxone. These prefilled syringes are also compatible with any method of administration, both nasal and injection routes. It is a recently approved IN naloxone formulation with a 4 mg/0.1 mL concentration. It is not yet widely available in the US.

In May 2023, the FDA approved Opvee®, a nasal spray version of nalmefene and the first alternative opioid antagonist indicated for opioid overdose reversal. The half-life of nalmefene is ~ 11 h, much longer than the 60- to 90-min half-life of naloxone [42]. Nalmefene may reverse opioid intoxication for longer than naloxone, which some view as a benefit over naloxone. However, its extended half-life presents the continued concern of placing opioid-dependent persons who overdose into precipitated opioid withdrawal for far longer than naloxone. Opvee was developed by Opiant Pharmaceuticals, which plans to release Opvee to the U.S. market as early as October 2023 [43]. Opiant also contributed to the development and manufacture of Narcan[44].

Bioavailability, referring to the proportion of a drug that is able to enter the body's circulation to have an active effect [45] and is a key consideration to take into account during discussions of naloxone dosing. It is used to approximate a drug’s effectiveness when taken by a patient. Factors that can affect bioavailability include the administration route (e.g. IN, IM, IV), drug form (e.g. tablet, liquid), and personal characteristics (e.g., age, weight, liver function).

The IN and IM administration routes are both commonly used by first responders in the community. They have bioavailability of 50% and 98% and a latency time to effectiveness of 15-min and 8-min, respectively [42, 45–47]. The IV route is only used in inpatient medical settings and has a bioavailability of 100% with a 2-min latency to effect. The IV route is preferred by medical professionals as the dose can be tailored to each patient allowing for sufficient overdose reversal while minimizing the risk of withdrawal.

## Methods

This study was conducted using the principles outlined by researchers with lived experience [38, 48]. Tennessee Harm Reduction is a drug-user run community-based organization in rural West Tennessee focused on distributing naloxone, drug checking supplies, and safer use supplies to over 200 community members. The organization’s Director (second author) and Outreach Specialist (first author) initially connected with the third and senior authors at the Harm Reduction Innovation Lab to share their experiences, knowledge, and perspectives surrounding high-dose naloxone formulations. Through their work, DPG and PLM found that PWUD reported that IN naloxone seemed to cause worse precipitated withdrawal compared to IM naloxone and that it seemed to take longer to reverse the overdose. As a result, people giving or receiving naloxone became cautious about continuing to use it. Tennessee Harm Reduction’s client base have reported over 215 known overdose reversals, the majority of which (135) used IM naloxone first. About half of those only needed one dose and a further 40% required two doses with the remaining 10% having 3 or more doses administered. No unsuccessful overdose reversals have been reported. It is important to note that both time between doses and whether the administrator had proper training was not reported so it is unclear whether 3 or more doses were actually needed. High-dose naloxone formulations caused even worse symptoms of precipitated withdrawal. DPG also noted that newly available high-dose naloxone formulations cost between 2 to 10 times more than generic IM naloxone.

Given the limited resources allocated to harm reduction organizations, we saw a need to better understand whether adopting high-dose naloxone formulations would be beneficial. Through an unfunded partnership between Tennessee Harm Reduction and the Harm Reduction Innovation Lab, a search strategy was implemented between August 2022 and February 2023. Phrases included: “high-dose naloxone formulation”, “opioid overdose”, “naloxone dosage overdose”, “naloxone dosage”, “high dose naloxone”, “high dose naloxone opioid”, “naloxone dosing”, “naloxone formulation”, “high dose naloxone formulation”, “high-dose naloxone”, and “high-dosage naloxone”. A literature review was performed using search engines PubMed and Google Scholar to compile a collection of relevant scholarly works. We filtered for original peer-reviewed articles published between January 2012 to February 2023 when heroin and fentanyl became the leading cause of U.S. overdose death. We also conducted a Google search to gather information on each naloxone product and cited research. We reviewed the title and abstract of each article and excluded those that focused on unrelated concepts.

The remaining eligible articles were summarized by research assistants using a matrix developed by the study team. We extracted article characteristics, including support or opposition to high-dose naloxone formulations, any funding received, the authors’ employment/conflicts of interest, main findings, if more than two doses of naloxone were administered, and the stated advantages/disadvantages of high-dose naloxone formulations. The findings were discussed and organized into three topics as described below.

Given the number of articles funded by naloxone manufacturers or consultants paid by pharmaceutical companies, we decided to remove those articles and discuss them in a separate section in order to minimize bias.

## Results

**Fig. 1:**
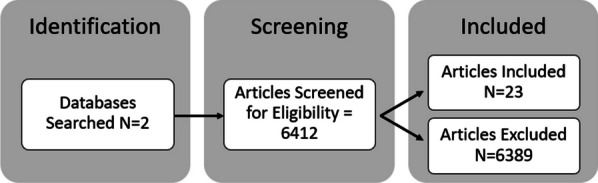
Flow chart of all articles included

We identified 23 articles eligible for inclusion (Fig. 1). Most articles were based on community-based response (N = 8) or medical response (N = 13) which included EMS response, hospitals, and outpatient settings. The 3 remaining articles focused on a combination of site types or police response. Most articles did not specify the brand of naloxone that was used but 2 explicitly focused on Narcan. The majority of the articles included multiple administration routes (N = 15) whereas 3 focused on intravenous, 1 on intramuscular, and 3 on intranasal. Two articles did not specify how naloxone was administered in the reported data. Additionally, no included articles noted whether oxygen was given to support overdose reversal and recovery despite that it has been shown to improve the success of naloxone [9].

We coded each article as supportive of high-dose naloxone formulations (N = 7; 30%), unsupportive (N = 4; 17%), or neutral (N = 12; 52%) as seen in Table 2. Notably, very few articles elicited the perspectives of PWUD. Six articles (25%) directly interviewed or surveyed PWUD. Common points of discussion identified in the articles included frequency of more than two doses, arguments supporting high-dose naloxone, and arguments against high-dose naloxone. Specific details of our synthesis are organized under these three overarching questions below.

**Table 2:** Published research articles involved in literature review

Overall Support of high-dose naloxone formulations	First author's last name, year of pub	Year of data collection	Jurisdiction	How often were more than two standard IN or IM doses needed	Evidence for high-dose naloxone formulations	Evidence against high-dose naloxone formulations
Yes	Abdelal, 2022	2020–2021	Select US Regions (with greater than 50% of opioids mortalities involving a synthetic opioid in 2018 (Northeast, Midwest or South, based on US Census Regions)	30% of participants (N = 125) used 3 or more doses of Narcan Nasal Spray (4 mg)	Authors conclude that high-dose naloxone formulations would be faster and fewer units may need to be carried if implemented but did not study high-dose naloxone formulations products	None mentioned
Yes	Bardsley, 2019	2017	Manchester, New Hampshire	Two clinical cases reported. Patient 1 required 6 doses of 2 mg/dose naloxone. patient 2 required 5 doses of 2 mg/dose naloxone. Both received IN and IV	Carfentanil exhibits greater affinity for μ-receptors than naloxone and may require high-dose naloxone formulations	Multiple doses can precipitate opioid withdrawal in overdose victims
Yes	Birmingham, 2017	2014–2016	Akron, Ohio	- Average dosing increased from 2 mg (Jan 2014) to 5 mg (Sept 2016) per patient. Unclear if IV, IM or IN naloxone was used	They did not study high-dose naloxone formulations products	None mentioned
Yes	Mahonoski, 2019	2015–2017	Maryland	Of 919 patients, 219 (24%) of patients required > 1 dose. 125/473 (26%) patients received 2 mg naloxone, 106/302 (35%) received 4 mg, 4/13 (21%) received 0.4 mg naloxone, and 12/29 (41%) received 1 mg naloxone to reverse opioid toxicity	The average naloxone dose increased from 2.12 mg to 3.63 mg, but clinical reversal rates decreased from 82 to 76%	Opioid withdrawal was reported in 12% of naloxone recipients. Other side effects included tachycardia, agitation, nausea and vomiting
Yes	Marco, 2018	2016–2017	Ohio	Unknown (dose per unit not provided)	82/89 patients were administered > 0.4 mg naloxone in combined prehospital and ED setting	Only 5/89 patients were administered naloxone at the ED setting, suggesting the possibility that higher doses were provided in the prehospital setting than necessary
Yes	Strickland, 2022	2021	US Addiction Treatment Facilities	60% of respondents used two or more naloxone doses	Among 1152 survey respondents, half had no high-dose naloxone formulations preference (48%) and about a third preferred to be revived using a high-dose naloxone formulations (35.9%)	None mentioned
Yes	Sutter, 2016	2016	Sacramento, California	7/18 patients given > 2 mg naloxone (route not specified), 4/18 received IV naloxone infusion. One patient died despite receiving 3 mg of naloxone	There are cases of respiratory depression recurrence after 8 h of observation without naloxone (longer than clinic’s protocol observation time)	None mentioned
No	Bell, 2018	2013–2016	Allegheny County, Pennsylvania	5% of cases required three or more doses of IM naloxone. Average dose of naloxone administered has not changed after the introduction of fentanyl	None mentioned	None mentioned
No	Carpenter, 2019	2017–2018	Atlanta, GA	Data from 121 patients. No difference in IV naloxone dose was found between fentanyl positive and opiate positive patients. Explanations include the purity of illicit fentanyl. More than 90% of overdoses were treated with concentrated formulations (IN and IM autoinjectors) but two doses of 0.4 mg IM injections would be insufficient in 40% of cases	None mentioned	These data refute idea that high-potency drugs require high-dose naloxone formulations
No	Farkas, 2021	2013–2016	Pittsburgh, Pennsylvania	359 patients. Total naloxone IV dose 3.2 ± 1.7 mg among patients who didn't need to be admitted to the hospital; 4.2 ± 2.5 mg among patients who were admitted	None mentioned	Additional naloxone administration by paramedics did not improve neuro-logic status but was associated with an increased likelihood of subsequent hospitalization. Clinically, other conditions that cause CNS depression must be addressed rather than using “open-ended escalation of naloxone therapy"
No	Hill, 2021	N/A	Literature review—US Centric	N/A	None mentioned	"There is little scientific evidence that supports the use of initial systemic dosing that exceeds 0.8 mg in the community.”
Neutral	Avetian, 2018	2016	US-Naloxone programs	Among 245 patients experiencing mostly heroin-suspected overdoses, three or more doses were needed 2% of the time	N/A	High antidote doses can induce precipitated withdrawal
Neutral	Faul, 2017	2012–2015	US-Nation wide	Multiple doses of naloxone increased 26% from 2012 to 2015, with increased odds of multiple doses in male patients Ages 20–50	Potential mismatch between typical naloxone dose and circulating opioids consumed by drug users. Because the half-life of naloxone is 30–90 min, multiple doses of naloxone may be needed when trying to reverse drug overdoses due to long-lasting opioids. Nasal passage scarring may reduce IN absorption	None given
Neutral	Klebacher, 2017	April 2014–June 2016	New Jersey	195 (9%) received two 2 mg IN doses (4 mg total) and 53 (2%) of patients received three doses of naloxone (6 mg total) by advanced life support. The rest received only one 2 mg dose	None mentioned	None mentioned
Neutral	Pourtaher, 2022	2015–2020	New York	9133 naloxone administrations by law enforcement from 2015 to 2020 involved an average of 2 doses of naloxone to aided individuals	Not mentioned	Not mentioned
Neutral	Purssell, 2020	2013–2017	Two Urban ED’s in Canada	Retrospective matched cohort study "…emergency department (ED) and ambulance service (EMS) records of opioid OD patients treated in two urban EDs serviced by a single EMS from Jan 1, 2013 to Dec 31, 2017 were reviewed." n = 80 LDN patients matched with 299 HDN patients"LDN was defined as an initial dose 0.15 mg naloxone and HDN as > 0.15 mg naloxone"	"At any given time after the initial dose of naloxone, HDN patients were more likely to have opioid reversal compared with LDN patients."	"HDN patients were more likely to have OW but also more likely to have opioid reversal versus LDN patients."
Neutral	Rzasa Lynn, 2018	N/A	International Lit Review	Recent reports of fentanyl and carfentanil toxicity resistant to naloxone reversal reflect a magnitude of over-dosing that results in an effect-site opioid concentration far exceeding that with which current standard doses of naloxone can compete for binding at the mu-opioid receptor	"However, in the hands of laypeople without adequate training or equipment to provide prolonged respiratory support, the risk of under-dosing naloxone far outweighs the potential risks of precipitating opioid withdrawal"	"Current evidence suggests that in the hands of trained medical personnel in an environment replete with additional life-support equipment, favoring the avoidance of withdrawal by utilizing a small initial dose of naloxone is safe"
Neutral	Schneider, 2021	November 2019–March 2020	Anne Arundel County, Maryland,	79.3% of N = 171 PWUD required three or more doses to reverse an overdose	No specific arguments made for high-dose naloxone formulations	None mentioned
Neutral	Somerville, 2017	October 1, 2014– March 31, 2015 [overdose data]. April 2016 [Interviews]	Barnstable, Bristol, and Plymouth counties in Massachusetts	Naloxone reverses overdoses involving IMF; multiple doses often required	Overdoses involving IMF are acute and rapid	None mentioned
Neutral	Tomassoni, 2017	June 2016	Yale New Haven Hospital, Connecticut	Unknown. The usual initial dose protocol is 0.1–0.2 mg IV. However, EMS was advised to increase their naloxone dosing during this outbreak	11/12 patients received > 2 mg naloxone (route: IN, IV, and/or IO). One patient received 0.5 mg total naloxone but died 3 days after admission. The outbreak was caused by fentanyl that had been sold as cocaine to an opioid-naive population, resulting in physiological susceptibility and/or difficulty recognizing opioid overdose	Unusual circumstances might not reflect naloxone doses required for the average opioid-familiar patient who might encounter similar doses of fentanyl
Neutral	Wong, 2019	November 2019–March 2020	No state or city specified- Population was two affiliated tertiary care ED’s in an urban city	First dose was between 0.4 mg and 2 mg of IV naloxone as per protocol. The study only included patients who needed additional doses.The role of take-home naloxone in the epidemic of opioid overdose involving illicitly manufactured fentanyl and its analogs. Fentanyl exposure was unmeasured	Similar adverse events between lower-dose and higher-dose groups (31% versus 41%, not sig): tachycardia, aggression/agitation, nausea/vomiting, abdominal discomfort/diarrhea, pulmonary edema. Inadequate dosing could occur if many improvised take-home naloxone kits that contain only two 0.4 mg doses for IM injection were solely deployed	"The lower end of the dosing range (e.g., 0.4 mg) appears to be a sufficient starting dose for opioid overdose in the ED and does not contribute to the recurrence of toxicity"
Neutral	Zuckerman, 2014	Not disclosed	Worcester, Massachusetts	26 y/o M. After 50 μg oral fentanyl, required 2 mg IN and 1 mg IV naloxone to regain consciousness, later needed additional 0.8 mg IV. Studies that review local medical examiner records) may miss nonlethal morbidity and read ministration of out of hospital naloxone	Current recommendations for staying with the patient “until they have woken up” underestimate the long duration of effect of commonly abused opiates, the short duration of effect of naloxone, and the risk of recurrence of apnea. Further medical care is needed. IN naloxone has unpredictable absorption	"Focus groups with IVDU report that they have a fear of precipitating “dope sickness” following administration of home naloxone [19]. These users report that they would likely redose themselves with opioid medications to treat such withdrawal symptoms. Such behavior can be lethal. Death from overdose increases dramatically following recovery from nonfatal overdose; opioid withdrawal triggered by intranasal naloxone may be a powerful motivator to reuse and cause more harm than good"
Neutral	Skulberg, 2022	Not disclosed	Oslo and Trondheim, Norway	Among 201 heroin-using patients, A commercially available 1.4 mg/0.1 mL IN naloxone restored breathing in 80% of cases compared with 97% among patients receiving 0.8 mg/2 mL of IM naloxone	None mentioned	None mentioned

### Part one: how often are more than two standard doses of im or in naloxone needed to reverse a fentanyl overdose?

Relatively few papers have been published examining the number of doses of IM or IN naloxone needed to reverse an overdose. Supporters of high-dose naloxone formulations often reference a single nationwide study conducted from 2012 to 2015 [49], which reported a trend of Emergency Medical Service (EMS) providers needing to administer more doses per patient each year. However, this study is limited in its methodology as it fails to consider administration route, dose volume, and dose concentration. The omission results in uncertainty that is driving the need for more doses, an important factor to understand before changing dosing practices.

Other researchers report that there was an increase in the average dose provided by EMS. One Ohio-based study from 2014 and 2016 reported the average IV dose increased from 2 to 5 mg [50]. However, a study conducted in Pittsburgh from 2013–2016 found that less than 5% of overdoses required three or more doses of IM naloxone [51]. This was corroborated by a national study from 2018, though heroin not fentanyl was involved in the majority of these cases [52]. Similarly, only two percent of EMS responders in New Jersey reported requiring a third dose of IN naloxone after having a second dose administered by an advanced life support team [53]. A large study of New York police officers from 2015–2020 noted that an average of two doses of naloxone were administered to rescue individuals [54]. One survey-based study reported that 30% of participants living in regions with fentanyl epidemics used 3 or more doses of IN naloxone [11]. A randomized double-dummy controlled trial found that the risk of receiving additional doses was 19.4% higher in those given IN naloxone (1.4 mg/0.1 mL) compared to IM (0.8 mg/2 mL), and that IN naloxone was less efficient in bringing overdose patients back to spontaneous breathing within 10 min in the prehospital setting[55]. However, heroin was the suspected drug in 196 of the 201 participants analyzed.

Interestingly, a Morbidity and Mortality Weekly Report (MMWR) reported 83% of patients in Massachusetts required 3 or more doses of nasal naloxone to reverse a suspected fentanyl overdose [56]. A 2021 survey-based study out of Maryland indicated that 79% of participants administered 3 or more doses at their last witnessed overdose [57]. Case studies and hospital chart reports have recorded high doses (12–15 mg) of naloxone being administered for synthetic opioid overdoses [58]. More than two doses of naloxone were required to reverse two carfentanil overdoses, likely owing to the greater affinity of carfentanil for μ-receptors than naloxone [59, 60]. μ-opioid receptors are one of the specific target sites in the body that naloxone binds to and blocks, effectively reversing the effects of opioid drugs [61, 62].

An aforementioned rigorous systematic review by Moe and colleagues of overdoses (n = 26,660) from North America and Europe through 2018 found that less patients with presumed fentanyl/ultra-potent opioid exposure were revived using initial low doses (≤ 0.4 mg/ml) versus when heroin was presumed (57% vs. 80%) but they concluded that a cumulative dose of 4 mg (e.g., two standard doses of IN) was sufficient in 97% of presumed fentanyl/potent opioid cases [29].

In conclusion, although there have been greater doses used in clinical and community settings, evidence suggests that the vast majority of fentanyl overdoses can be reversed with standard dosing. However, overdoses involving carfentanil or other similarly potent synthetic analogs may require three or more doses. Two doses of IM naloxone (0.8 mg) have also been insufficient in reversing some fentanyl overdoses though such data are subject to the amount of fentanyl exposure [63]. Additionally, depending on the half life of the opioid used, an individual may fall back into an overdose after being revived due to naloxone’s half life of 30–90 min [64]. Given these facts, and the observation that three or more doses are already being used in the community, our recommendation is that four doses of IN or IM naloxone be provided to community members with clear education on the length of latency to effectiveness to optimize coverage. To determine whether high-dose naloxone formulations are an optimal solution we weigh the advantages and disadvantages of these formulations next.

### Part two: what are the potential advantages of high-dose formulations?

There are many perceived benefits of high-dose naloxone formulations, though, in practice, the evidence base is underdeveloped. Given that the formulations currently approved have been relatively comparable in their concentration to others of the same administration route, much of the literature has focused on administering naloxone slowly over time (“titration”) rather than administering a single high-dose naloxone formulation. According to the small number of papers [46, 65] on this topic, a high-dose naloxone formulation could theoretically result in a faster response and reduce the magnitude of the harmful non-fatal impacts of drug toxicity, including cognitive and physiologic issues, although this was not proven with real-world data. A high-dose naloxone formulation would improve reversal rates for overdoses involving carfentanil and other opioids that have a stronger μ-receptor affinity than fentanyl [59], though such experiences are relatively rare and localized. Interestingly, recent national study showed that almost half (48%) of people who had reversed an overdose with naloxone held no preference for, or were against, high-dose naloxone formulations, while over one third (36%) preferred a high-dose naloxone formulations to be made available [37].

Owing to the preconceived biases around drug use and naloxone by association, the need to carry fewer doses may help reduce experiences of stigma [11], while simultaneously providing more convenience in portability. Despite these potential benefits and community interest in high-dose naloxone formulations [37], we see many potential pitfalls of relying solely on them.

### Part three: what are the potential disadvantages of high-dose formulations?

As discussed previously, prior literature suggests that, despite fentanyl poisoning becoming more prevalent, a standard dose can still be equally effective in many cases [36, 51, 66–68]. Below, we discuss further reasoning to not recommend high-dose naloxone formulations over standard dosing.

#### There is no pharmacological basis for high dose naloxone when it comes to fentanyl

In vivo, in the human brain, researchers have used a positron emission tomography (PET) scanner’s non-tomography positron detecting system to measure the dose–response curve of naloxone and found that ~ 13 μg/kg (0.013 mg/kg) of naloxone per kg of patient bodyweight was required to produce an estimated 50% receptor occupation when given intravenously[69]. In general, a drug typically needs to occupy a sufficient number of its target receptors to initiate the desired biological response. Studies have indicated that achieving approximately 50% receptor occupancy by naloxone is associated with its desired therapeutic effects in reversing opioid overdose [69, 70]. This suggests that higher doses of naloxone may not be needed as long as 50% of the μ-opioid receptors are occupied. However, there are many factors that could affect level of occupancy such as route of administration and bioavailability [71].

A second pharmacokinetic consideration is the variable binding affinities of various opioids relative to the antagonistic effects of naloxone. Each opioid has a unique binding affinity (K_i_) towards the μ-opioid receptors. Naloxone must have a lower K_i_, indicating a stronger binding affinity, to successfully reverse an overdose. Notably, morphine and fentanyl have similar K_i_ values despite their vastly different potency levels demonstrating that potency does not always correlate with binding affinity [72, 73]. Therefore, stronger analogs are not an indication of the need for high-dose naloxone formulations in the absence of binding affinity assessment.

While naloxone exhibits a relatively rapid and strong binding affinity to opioid receptors, the short duration of action means it is relatively quick to dissociate from the receptors [70, 74]. For longer acting opioids, this means opioids may rebind causing a recurrence of respiratory depression. high-dose naloxone formulations have been found to make the effect last longer but doesn't change how quickly it works to reverse an overdose[75]. The duration of a drug's effects can vary depending on several factors, including the individual's tolerance, the method of administration, the dosage, and the purity of the drug. high-dose naloxone formulations may have an application for these longer-acting opioids and circumstances, but evidence is limited.

#### The risk of withdrawal from high-dose naloxone

As with any medication, there are potential risks associated with taking too much naloxone. The main risk of excessive naloxone dosing is that it can cause rapid-onset naloxone-induced withdrawal symptoms if a person has a high dependence to opioids [76–78]. Naloxone is effective at reversing overdose as it displaces opioids from the receptors without activating their sedative and respiratory depressant effects. The displacement effectively reverses the effects of the opioids and causes withdrawal even if opioids remain in the person's system [79, 80]. This can include the well-known symptoms such as severe pain, agitation, muscle cramps, and nausea [81]. Additionally, precipitated withdrawal has serious symptoms, such as diarrhea, vomiting, myalgia, anxiety, and autonomic hyperactivity [82]. Additionally, in rare cases, naloxone can cause an allergic reaction, such as hives, difficulty breathing, or swelling of the face, lips, tongue, or throat [5]. Sequelae such as death, coma, and encephalopathy have been documented in association with these occurrences. Notably, such events have predominantly manifested in patients with pre-existing cardiovascular disorders or those concurrently administered medications with comparable adverse cardiovascular effects. However, establishing a definitive cause-and-effect relationship requires further investigation [83].

Due to these risks, the recommended dose for opioid reversal remains controversial. The aforementioned withdrawal risk can lead to hesitation among PWUD when encountering a potential overdose. They must quickly balance the potentially life threatening consequence of withholding the narcan with the ensuing implications of withdrawal. Namely, that the intense discomfort and cravings following withdrawal can lead to subsequent increased use and opioid seeking behaviors. Additionally, negative experiences related to overdose reversal may result in avoidance of treatment due to fears of having similar experiences in an already stressful medical setting. Finally, withdrawal can temporarily impair an individual's ability to carry out acts of daily living such as caring for oneself, attending work, or engaging in social activities. high-dose naloxone formulations are likely to intensify these drawbacks as administration to someone with high opioid dependence can lead to more intensified symptoms then the typical dose would as more opioids will be displaced with naloxone.

#### The need for respect, consent, and a voice in drug policy: ethical considerations

From a literal perspective, consent is the act of voluntarily agreeing to participate in something, such as a medical procedure, sexual activity, or research study. It is important because it ensures that individuals know and understand what they are agreeing to. Consent is a fundamental aspect of respecting individual autonomy and personal freedom and it is crucial for maintaining ethical standards in healthcare, research and interpersonal relationships.

In the context of an overdose, obtaining consent is not possible because the victim is unconscious. Unless the responder and person experiencing the overdose had discussed their preferences on how to handle such a situation before the overdose occurred, standard guidance should aim to do as little harm as possible. In such cases, it's also important to provide clear and accurate information and to respect their autonomy as much as possible after administration of naloxone when conscious.

Listening to PWUD is crucial in making informed decisions about increasing the dose formulation of naloxone. Those with lived and living experience have unique insight into the complexities of overdose and the effectiveness of naloxone. They can provide valuable information on how a higher dose formulation may impact their ability to respond to an overdose. Additionally, they can offer insight into other factors that may contribute to overdose, such as polysubstance use or lack of access to harm reduction services. By listening to those with lived and living experience, we can gain a better understanding of the challenges and barriers faced by PWUD and make more informed decisions about how to address overdose in a way that is effective, equitable, and inclusive.

Collaborating with PWUD is also an important aspect of practicing informed consent. By actively hearing their experiences and concerns, we can better understand their needs and preferences allowing us to provide care with respect and consideration of their unique circumstances. Individuals who use drugs have the right to make informed decisions about their healthcare and incorporating their preferences ensures they are empowered to make informed decisions about their healthcare. This can help build trust between healthcare providers and PWUD, leading to better health outcomes and more effective overdose prevention strategies. Conrarily, making decisions on naloxone dose, route of administration, and cost without including those who are directly impacted in the decision process violates their right to consent, erodes their trust and perpetuates the overdose epidemic.

#### Cost considerations

As shown in Table 1, the costs of available naloxone formulations vary widely from $15-$40 per unit for the most affordable generic IM formulation to $131-$145 per unit for Zimhi high-dose IM auto injector and Kloxxado high-dose IN. As expected, generic formulations cost less than branded formulations with the IM and IN costing $15 and $20 at the lower cost range respectively. Notably, the two highest single dose formulations, Zimhi and Kloxxado, are also the most costly. Zimhi is 25 times stronger than generic IM naloxone and costs over 8 times the generic equivalent. Kloxxado is twice as strong as the generic IN formulation and costs about 5 times as much. Given that the majority of fentanyl overdoses studied only require two or three doses of standard IM or IN, high dose naloxone formulations with more than three times the dose within the same administration route category may not be a cost effective solution.

## Discussion

We aimed to understand whether two doses of IM/IN naloxone can effectively reverse fentanyl overdoses and whether newer high-dose formulations are an optimal and necessary solution. Our findings indicate that although two or more standard doses of naloxone have been administered in clinical and community settings, most fentanyl overdoses can be successfully reversed using two standard dosages of IN or IM. Overdoses involving carfentanil, a highly potent fentanyl analog, necessitate three or more doses for effective reversal; this may be due to carfentanil having a slower rate of opioid receptor dissociation [84]. However, carfentanil overdoses are relatively rare compared to fentanyl overdoses throughout the United States.

Although comparing formulations was beyond the scope of our review, we did note that in some cases, the administration of two IM naloxone doses (0.8 mg) has been insufficient in reversing a fentanyl overdose. However, the accuracy of this conclusion is contingent upon the quantity of fentanyl present in the drug samples consumed and the individual’s tolerance. For this reason, community-based programs that solely distribute IM naloxone could pre-emptively begin distributing four or more doses to all program participants. Given the well-established knowledge that overdose symptoms may recur after resuscitation, depending on the half-life of the specific opioid, keeping additional doses of naloxone on hand can be useful regardless of the formulation distributed.

Considering these findings and the current community practice of using multiple doses of standard IM and IN, we recommend providing, at minimum, four standard doses of IN or IM naloxone to each individual (i.e., two two-dose kits). This guarantees that administration can continue until the recipient achieves stability, ensuring appropriate intervals between each dose, and extra doses are on hand in case of carfentanil exposure or symptom recurrence. Given that some people who use fentanyl use multiple times per day, and some bystanders know multiple people who use fentanyl, providing an ample number of kits to potential bystanders is critical.

Higher-dosage formulations are unnecessary for fentanyl overdoses, and may also cause harm as evidenced by the risk of precipitated opioid withdrawal. While there is little evidence that high-dose naloxone formulations will be more effective for responding to fentanyl overdoses, high-dose naloxone formulations may elicit a faster overdose reversal rate for carfentanil overdoses compared to standard doses.

One barrier that remains in scaling up IM and IN naloxone is that only one brand of over-the-counter IN naloxone (Emergent) has been FDA approved. Approving generic naloxone and standard IM formulations will help speed up community-level naloxone coverage. Another barrier to carrying IM naloxone is that syringe possession remains illegal in some states.

### Data limitations

Much of the literature supporting the use of high-dose naloxone formulations fails to take into consideration the expressed needs, barriers, and consent of PWUD, which may have significant implications for the ethical and effective implementation of such interventions. For these reasons, we encourage scientists, medical providers, and pharmaceutical companies to speak to PWUD and service providers (such as harm reduction workers or others working directly with drug users) when developing and testing new high-dose naloxone products. Providing the context to epidemiological and clinical data through lived experience is important because it allows for a more accurate interpretation of the results as well as a more realistic understanding of how naloxone formulary changes would impact PWUD. Without context, assumptions may be based on bias, or draw the wrong conclusions. We also noted that some studies were either conducted or funded by pharmaceutical companies who may have a conflict of interest in the study’s outcome.

### Future studies and conclusions

The majority of the research conducted in the field of substance use has not been done in settings that accurately reflect the contexts in which PWUD experience an overdose and withdrawal symptoms. For example, there has been scientific debate on the role of non-opioid sedatives such as xylazine (a tranquilizer commonly used in veterinary medicine) and benzodiazepines (a central nervous system depressant) in overdose response[85–87]. We must communicate to the public that naloxone will not reverse the effects of these sedatives and additional medical intervention may be required to assist PWUD even after naloxone is administered. More studies that center the perspectives of PWUD are needed to optimize community bystander reversals especially in the era of xylazine and other contaminants.

In conclusion we did not find rigorous evidence to support the distribution of high-dose naloxone formulations compared to standard doses. Community programs should provide at least four doses of standard IM or IN (and more if possible) to each program participant to optimize naloxone coverage without sacrificing the physical and psychological wellbeing of PWUD.

## Data Availability

Data sharing is not applicable to this article as this is a literature review. However, Table 2 has been provided that lists all published research articles involved in the literature search for this review. The data referenced by Tennessee Harm Reduction are not publicly available because it was used only as observation. However, a summary of this data is available on the Tennessee Harm Reduction website and data from the corresponding author on reasonable request.

## Abbreviations

CPR: Cardiopulmonary resuscitation
EMS: Emergency medical services
FDA: Federal drug administration
IM: Intramuscular
IN: Intranasal
IV: Intravenous
K_i_: Binding affinity
MMWR: Morbidity and mortality weekly report
PET: Positron emission tomography
PWUD: People who use drugs
SAMHSA: Substance abuse and mental health services administration
SUD: Substance use disorder
